# Knockdown of MAPK14 inhibits the proliferation and migration of clear cell renal cell carcinoma by downregulating the expression of CDC25B

**DOI:** 10.1002/cam4.2795

**Published:** 2019-12-19

**Authors:** Junlong Liu, Xiuyue Yu, Hongyuan Yu, Bitian Liu, Zhe Zhang, Chuize Kong, Zhenhua Li

**Affiliations:** ^1^ Department of Urology The First Hospital of China Medical University Shenyang P. R. China; ^2^ Department of Urology Shengjing Hospital of China Medical University Shenyang P. R. China

**Keywords:** CDC25B, clear cell renal cell carcinoma, DNA damage and repair, MAPK14, P‐MAPK14

## Abstract

Mitogen‐activated protein kinase 14 (MAPK14), which plays an important role in DNA damage and repair, is activated by various environmental stress and proinflammatory cytokines. It is highly active in a variety of tumors, acting as a tumor promoter or suppressor, but its role in clear cell renal cell carcinoma (ccRCC) has not been elucidated. Cell division cycle 25B (CDC25B) is involved in cell cycle regulation and is highly expressed in many malignant tumors. The transcription levels of MAPK14 and CDC25B in 72 pairs of ccRCC and adjacent healthy tissues from the cancer genome atlas database and the protein expression levels in 66 pairs of clinical samples were analyzed in this study. After MAPK14 was knocked down by small interfering RNA (siRNA), P‐MAPK14 and CDC25B protein levels decreased. Subsequently, Western blot and co‐immunoprecipitation demonstrated that P‐MAPK14 could bind to CDC25B, potentially maintaining its stability. The proliferation and migration of ccRCC cell lines were suppressed by siRNA knockdown of MAPK14, however, that could be partially reversed by the overexpression of CDC25B. These results suggest that downregulation of MAPK14 and P‐MAPK14 could inhibit the proliferation and migration of ccRCC by downregulating CDC25B.

## Graphical abstract

We examined the expression of mitogen‐activated protein kinase 14 (MAPK14) and cell division cycle 25B (CDC25B) in clear cell renal cell carcinoma (ccRCC) and healthy tissue using the cancer genome atlas database and clinical samples. We affirm that our results reveal that phosphorylated MAPK14 (P‐MAPK14) and CDC25B are highly expressed in ccRCC tissue, and that P‐MAPK14 binds to CDC25B, potentially stabilizing it. Additionally, MAPK14 knockdown inhibits the proliferation and migration of ccRCC cells, an effect that is partially reversed by CDC25B overexpression, suggesting that downregulation of MAPK14 and P‐MAPK14 inhibits the proliferation and migration of ccRCC by downregulating CDC25B.

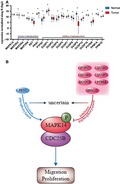

**Abbreviations**
CDC25B: cell division cycle 25B
IHC: immunohistochemistry
MAPK14: mitogen‐activated protein kinase 14
P‐MAPK14: phosphorylated mitogen‐activated protein kinase 14
RCC: renal clear cell carcinoma

## 1 INTRODUCTION

Renal cell carcinoma (RCC) is the ninth most common cancer in the world and is therefore a major public health concern worldwide.^1^ The American Cancer Society projects that in 2019, 73,820 people will be diagnosed with kidney and renal pelvic cancer and 14,770 of those diagnosed will die.^2^ The occurrence and metastasis of RCC are extremely complicated, as they are not only related to genetic mutations but also to the imbalances in the tumor suppression and activation pathways.^3^ Clear cell RCC (ccRCC) is the most common and aggressive subtype of RCC, accounting for approximately 70% of all the cases.^1^, ^4^ Surgery is still the major therapeutic method for patients with localized ccRCC, and metastatic lesions are resistant to chemotherapy and radiotherapy at the time of initial diagnosis.^5^, ^6^ Although great progress has been made in the targeted treatment of metastatic RCC in recent years, the progression‐free survival is still not satisfactory.^7^ Therefore, it is necessary to identify the effective biomarkers and new therapeutic targets for ccRCC in order to improve the prognoses of these patients.

The mitogen‐activated protein kinase (MAPK) signaling pathway plays an important role in cell integration and is activated in response to a variety of environmental and cellular stresses, inflammation, and other signals.^8^, ^9^ Four different p38 MAPK have been identified, each encoded by a single gene: p38α (MAPK14), p38β (MAPK11), p38γ (MAPK12), and p38δ (MAPK13). The majority of p38 MAPK functions are carried out by MAPK14 (also known as p38α).^10^, ^11^ Recent studies have shown that MAPK14 is highly expressed in many tumors. Moreover, it can promote the occurrence, invasion, and migration of tumor cells, such as breast cancer, ovarian serous adenocarcinoma, and gastric cancer.^12^, ^13^, ^14^ However, the biological function of MAPK14 in ccRCC has not been elucidated.

Cell division cycle 25B (CDC25B) is a member of the CDC25 phosphatase family which consists of three members. The other two subtypes are CDC25A and CDC25C, both of which are crucial in cell cycle regulation.^15^ CDC25B is activated in the G2/M phase of the cell cycle and is highly expressed in many malignant tumors.^16^, ^17^ It has been shown that CDC25B is involved in the activation of CDC2 in the G2/M phase by dephosphorylating CDC2,^18^ and knockdown of CDC25B can block the cell cycle and inhibit cell proliferation in the G2/M phase.^19^, ^20^ Additionally, p38 MAPK has been shown to bind to and phosphorylate CDC25B, which is critical for G2/M checkpoint initiation.^21^ Therefore, CDC25B phosphatase is a potentially attractive target for the management of ccRCC.

In this study, it was revealed that phosphorylated MAPK14 (P‐MAPK14, Thr180, and Tyr182) and CDC25B were overexpressed in ccRCC, and P‐MAPK14 might affect the stability of CDC25B. Moreover, MAPK14 might promote the proliferation and migration of ccRCC both in vivo and in vitro, probably by stabilizing the expression of CDC25B protein.

## 2 MATERIALS AND METHODS

### 2.1 The cancer genome atlas (TCGA) data

TCGAbiolinks is a program used to facilitate the retrieval and processing of data from the open‐access GDC Data Portal. From the preprocessed Kidney Renal Clear Cell Carcinoma or KIRC data, 72 pairs of cancer samples with adjacent healthy samples were screened to analyze the target gene and related genes.

### 2.2 Tissue specimens and clinical data collection

The tissue samples used in this study (tumor and adjacent healthy tissues) were obtained from 66 patients admitted to the First Hospital of China Medical University (Shenyang, China) from January 2015 to December 2018. All tissues were examined histologically and diagnosed as ccRCC. The Ethics Committee of the First Hospital of China Medical University approved this study according to the Declaration of Helsinki, and written informed consent was obtained from all the patients. Tissue samples were stored at −80°C prior to use.

### 2.3 Cell lines and cell culture

The human ccRCC cell lines, ACHN and CAKI‐1, were obtained from the Chinese Academy of Sciences Type Culture Collection Cell Bank (Shanghai, China). The cells were cultured according to the manufacturer's guidelines and passage time was less than 6 months with mycoplasma eliminated. ACHN cells were cultured in MEM medium (HyClone, Logan, UT, USA) and CAKI‐1 cells were cultured in McCoy's 5A medium (HyClone, Logan, UT, USA), both media were supplemented with 10% fetal bovine serum (FBS, HyClone). All ccRCC cell lines were cultured in a humidified atmosphere of 5% CO_2_ at 37°C. When the cell fusion rate was 90%, the cells were digested with 1‐ml trypsin for 5 minutes, neutralized with 1 ml of medium, collected by centrifugation, and passaged.

### 2.4 Small interfering RNA (siRNA) and plasmid transfection

ACHN and CAKI‐1 cell lines were cultured in 6‐well plates. When the confluence of the cells was 60%, they were transfected with either MAPK14 siRNA or CDC25B siRNA (JTS scientific, Wuhan, China) using Lipofectamine™3000 (Invitrogen, USA), according to the manufacturer's guidelines. The following siRNA sequences were used (5′‐3′): siMAPK14‐1# (senseCCAGACCAUUUCAGUCCAUTT3 and anti‐senseAUGGACUGAAAUGGUCUGGTT3), siMAPK14‐2# (sense CCUUGCACAUGCCUACUUUTT and anti‐sense AAAGUAGGCAUGUGCAAGGTT), siCDC25B‐1# (sense GCAACAUCGUGGAUAAGUUTT and anti‐sense AACUUAUCCACGAUGUUGCTG3), siCDC25B‐2# (sense GAGAGCUGAUUGGAGAUUATT and anti‐sense UAAUCUCCAAUCAGCUCUCGG) siNC (sense UUCUCCGAACGUGUCACGUTT and anti‐sense ACGUGACACGUUCGGAGAATT).

MAPK14 knockdown and CDC25B overexpression plasmids were synthesized by and purchased from GeneChem (Shanghai, China). The stability of ACHN and CAKI‐1 cells with overexpressed CDC25B was screened over 4 weeks using G418 (400 μg/ml). The stability of ACHN‐downregulated MAPK14 cells was analyzed over approximately 4 weeks using puromycin (2 μg/ml).

### 2.5 RNA extraction and RT‐PCR

Total RNA was extracted from ACHN and CAKI‐1 cells 48 hours after siRNA transfection. RNAiso Plus (Takara Biotechnology, Dalian, China) was used according to the manufacturer's protocol. cDNA was generated using the PrimeScript™RT Master Mix (Takara, Dalian), and RT‐PCR was performed using the SYBR premix ExTaq™ kit (Takara, Dalian). Detection was performed using a LightCycler™480 II system (Roche diagnostics, Switzerland). GAPDH was taken as the internal parameter. Primers for MAPK14, CDC25B, and GAPDH were as follows (5′‐3′): MAPK14 (sense CCCGAGCGTTACCAGAACC and anti‐sense TCGCATGAATGATGGACTGAAAT), CDC25B (sense ACGCACCTATCCCTGTCTC and anti‐sense CTGGAAGCGTCTGATGGCAA) and GAPDH (sense ACAACTTTGGTATCGTGGAAGG and anti‐sense GCCATCACGCCACAGTTTC). The $2^{-\Delta \Delta C_{t}}$ method was used to calculate the relative expression of different genes.

### 2.6 Western blot

Total tissue and cell lysates were extracted from the radioimmunoprecipitation test buffer containing protease and phosphatase inhibitors. The protein concentration was determined by bicinchoninic acid assay (Beyotime Institute of Biotechnology). Denatured protein (40 μg/lane) was separated on a 10% sodium dodecyl sulfate polyacrylamide gel electrophoresis (140 V, 50 minutes) gel, followed by transfer to a polyvinylidene fluoride membrane (350 mA, 90 minutes). The membrane was blocked in a sealed container using 5% fat‐free milk at 37°C for 1 hour. The membrane was incubated overnight with the primary antibody in 5% fat‐free milk at 4°C. The primary antibodies were as follows: anti‐MAPK14 (1;1000, 9218, CST), anti‐P‐MAPK14 (1;1000, 4511, CST), anti‐CDC25B (1;100, ab70927, abcam), anti‐P‐CDC2 (1:1000, ab47594, abcam), anti‐E‐Cadherin (1:10 000, ab40772, abcam), anti‐N‐Cadherin (1:5000, ab76011, abcam), and anti‐β‐tubulin (1:1000, 2128S, CST). The membrane was washed three times (5 minutes each) using tris‐buffered saline tween‐20, followed by incubation with the corresponding secondary antibody at 37°C for 1 hour. EasySee Western Blot kit (Beijing Transgen Biotech, Beijing, China) was used for detection according to the manufacturer's guidelines. Density measurements were carried out using ImageJ (National Institute of Health, Bethesda, MD, USA), with the protein bands normalized to β‐tubulin.

### 2.7 Cell viability assay

Cell viability was measured by colorimetric assay using Cell Counting Kit‐8 (CCK‐8) (Bimake, USA). Cells were seeded in 96‐well plates at 3 × 10^3^ cells per well. After 24 hours of transfection, CCK‐8 solution was added to the cells to a final concentration of 0.5 mg/ml and incubated at 37°C for 1 hour. Absorbance at 450 nm was measured using a plate reader (Model 680; Bio‐Rad Laboratories).

### 2.8 Cell proliferation assay

ACHN and CAKI‐1 cells were transfected with MAPK14 siRNAs in 24‐well plates for 48 h, with EdU (BeyoClick™, EDU‐488, China) added to the medium (1:1000) according to the manufacturer's guidelines. Cells were cultured for 2 hours at 37°C, after labeling, the culture medium was removed, and 1‐ml fixative solution (4% paraformaldehyde) was added at room temperature for 20 minutes. Cells were incubated at room temperature for 15 minutes with 1‐ml permeate (0.3% Triton X‐100) and the click reaction buffer was added according to the manufacturer's protocol. A fluorescence microscope (Olympus Corporation, Japan) was used to obtain the images.

### 2.9 Transwell assay

Transfected cells (1 × 10^5^) in 200‐μl serum‐free medium were inoculated on the upper chamber (Corning, NY, USA) of the 24‐well plates, and 600 μl of the medium (10% FBS) was added to the bottom of the chamber. Cells were incubated at 37°C and 5% CO_2_ for 24 hours. A swab was used to remove any remaining cells from the upper chamber, 4% paraformaldehyde was used to fix the cells for 10 minutes, and crystal violet stain was added for 10 minutes. Cell migration was detected using optical microscopy, and ImageJ was used to calculate the migration efficiency.

### 2.10 Co‐immunoprecipitation

Cells were harvested in a culture dish and the appropriate amount of cell lysate was added (including protease and phosphatase inhibitor). Cells were lysed on ice for 30 minutes, followed by centrifugation at 1.2 × 10^4^ g for 30 minutes, and the supernatant was removed. A small amount of pyrolysis liquid was used for Input group, a primer antibody P‐MAPK14 or IgG (Santa Cruz Biotechnology) was added to residual cracking liquid, and 30‐μl protein A/G‐beads was added to the cell lysis solution and left slowly shaking at 4˚C overnight. The beads were then washed three times with 1‐ml pyrolysis buffer, followed by 20 μl of 2 × protein loading buffer at 100°C for 10 minutes. Western blot was then performed.

### 2.11 Tumor formation in nude mice

Four‐week‐old female nude mice were purchased from Beijing Vital River Experimental Animal Technology (Beijing, China). The Ethics Committee of Medical Experimental Animal Welfare of China Medical University approved this study, and the study was allowed to be performed at the Experimental Animal Department of China Medical University. The mice were divided into three groups with six mice for each group. Fifty microliter PBS containing 1 × 10^6^ treated ACHN cells (MAPK14‐KD, CDC25B‐OE, and Vector) and 50‐μl basilar membrane matrix were subcutaneously injected into the shoulder to establish a human ccRCC cell xenograft model. The mice were euthanized 28 days after injection, then the tumors were excised.

### 2.12 Immunohistochemistry (IHC)

IHC was performed on paraffin‐embedded xenograft tissues. Immunostaining was performed according to the manufacturer's guidelines using the avidin‐biotin‐peroxidase complex method (MaiXin, Fuzhou, China). Peroxidase inhibitors were used to block endogenous peroxidase activity. Sections were incubated with normal goat serum for 30 min to reduce nonspecific binding, followed by incubation with Ki‐67 antibody (MaiXin, Fuzhou, China) overnight at 4°C. Images were obtained using an optical microscope. The proportion of Ki‐67‐positive cells was counted and the proliferation index was determined.

### 2.13 Protein causal relationship

SIGNOR (signor.uniroma2.it), the Signaling Network Open Resource, organizes and stores information on signaling pathways, which has been published in the scientific literature. At the heart of the project is a collection of artificially annotated causal relationships between more than 11,000 participating proteins. The association was related to the experimental evidence reported in the literature. We used SIGNOR to find related proteins that might promote or inhibit MAPK14 phosphorylation.

### 2.14 Statistical analysis

GraphPad Prism version 7.0 (La Jolla, CA, USA) was used for statistical analyses. For comparisons, Mann‐Whitney *U*‐test, Pearson Chi‐square test, Pearson correlation analysis, and Student's t test were performed as instructed. Each experiment was performed independently at least three times (n ≥ 3). All data were expressed as mean ± SD *P* < .05 was considered statistically significant.

## 3 RESULTS

### 3.1 Expression and correlation of MAPK14, P‐MAPK14, and CDC25B in ccRCC and adjacent healthy tissue

**Figure 1:**
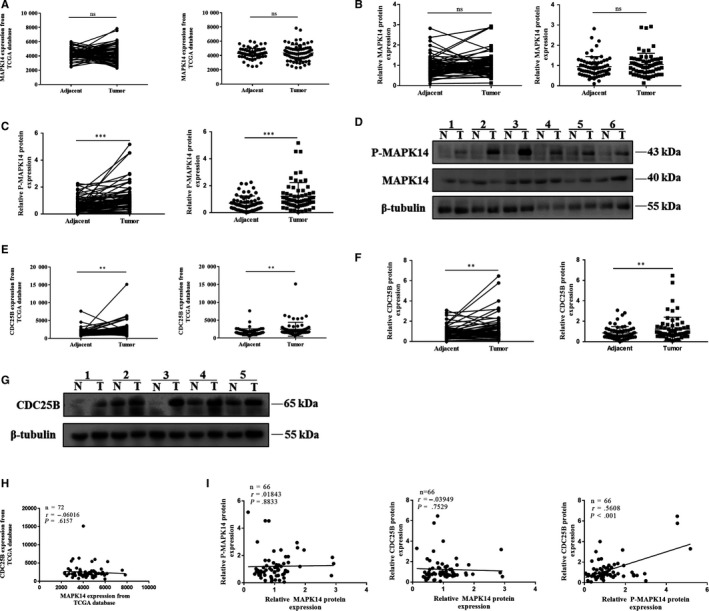
Expression and correlation of MAPK14, P‐MAPK14, and CDC25B in ccRCC and adjacent healthy tissue. A, MAPK14 mRNA levels in 72 pairs of ccRCC and adjacent healthy tissues from TCGA database (n = 72). B, D, MAPK14 protein levels in 66 pairs of ccRCC and adjacent healthy tissues detected by Western blot (n = 66). C, D, P‐MAPK14 protein levels in 66 pairs of ccRCC and adjacent healthy tissues detected by Western blot (n = 66). E, CDC25B mRNA levels in 72 pairs of ccRCC and adjacent healthy tissues from TCGA database (n = 72). F, G, CDC25B protein levels in 66 pairs of ccRCC and adjacent healthy tissues measured by Western blot (n = 66). H, Correlation of MAPK14 and CDC25B mRNA in 72 cases of ccRCC from TCGA database (n = 72). I, Correlation of MAPK14, P‐MAPK14, and CDC25B proteins in 66 cases of ccRCC. T tumor, N adjacent healthy tissue. *P* < .05 was considered significant (*P < .05, **P < .01, ***P < .001), ns, not significant

The transcription levels of MAPK14 in 72 pairs of ccRCC and adjacent healthy tissues were analyzed from TCGA database. No significant differences were observed (*P* > .05) (Figure 1A). MAPK14 protein expression in 66 pairs of clinical samples of ccRCC was detected by Western blot. These results revealed no significant differences (*P* > .05) (Figure 1B,D), consistent with the results from TCGA database. Next, the levels of P‐MAPK14 protein present in ccRCC and adjacent healthy tissues were determined. Our results revealed that there was a significant increase in the expression of P‐MAPK14 protein in ccRCC tissues in comparison to healthy tissues (*P* < .001) (Figure 1C,D).

CDC25B is a major member of the CDC25 family and is important for mitosis. Recent studies have shown that high expression levels of CDC25B can promote the malignant transformation of cells and promote tumor growth. Thus, the transcription levels of CDC25B in 72 pairs of ccRCC and adjacent healthy tissues from TCGA database were analyzed. The result revealed that CDC25B transcription level in ccRCC was significantly higher than that in adjacent healthy tissues (*P* < .01) (Figure 1E). CDC25B protein expression was also significantly higher in ccRCC tissues, as demonstrated by Western blot (*P* < .01) (Figure 1F,G). Western blot of all tissue images can be viewed in Figure S1.

Next, the correlation between MAPK14 and CDC25B mRNA in 72 ccRCC samples from TCGA database was analyzed, and no significant correlation was found (*P* > .05) (Figure 1H). Similarly, there was no correlation between MAPK14 and P‐MAPK14 or CDC25B at the protein level (*P* > .05). However, the expression of P‐MAPK14 was significantly correlated with CDC25B (*P* < .001) (Figure 1I).

To assess the significance of P‐MAPK14 and CDC25B expressions in ccRCC, the associations between P‐MAPK14 or CDC25B and the clinical characteristics were evaluated in ccRCC tissues (Table 1). P‐MAPK14 and CDC25B were not correlated with gender, age, or tumor stage. However, they were significantly correlated with pathological grading. In this study, it was not able to examine the correlation with distant metastases and lymphatic metastases because of the lack of advanced tumors.

**Table 1:** Correlation between P‐MAPK14 and CDC25B expression and the clinicopathological parameters of 66 ccRCC patients

Variables	Group	N	P‐MAPK14		*P*‐value^a^	CDC25B		*P*‐value^a^
			Low	High		Low	High	
Gender	Male	48	25	23	.5804	24	24	>.99
	Female	18	8	10		9	9	
Age	≥60	29	14	15	.8041	12	17	.7779
	<60	37	19	18		21	16	
T stage	T1/T2	64	32	32	>.99	32	32	>.99
	T3/T4	2	1	1		1	1	
Fuhrman grade	1/2	48	29	19	.0057*	28	20	.0270*
	3/4	18	4	14		5	13	
Distant metastasis	Negative	64	32	32	>.99	31	33	.1510
	Positive	2	1	1		2	0	
Lymphatic invasion	Positive	0		–				–

a *P*‐value was determined by Pearson Chi‐square tests and ^*^ *P* < .05 was considered significant.

### 3.2 Knockdown of MAPK14 resulted in decreased expression of P‐MAPK14 and CDC25B while overexpression of CDC25B did not affect MAPK14 and P‐MAPK14 expressions

**Figure 2:**
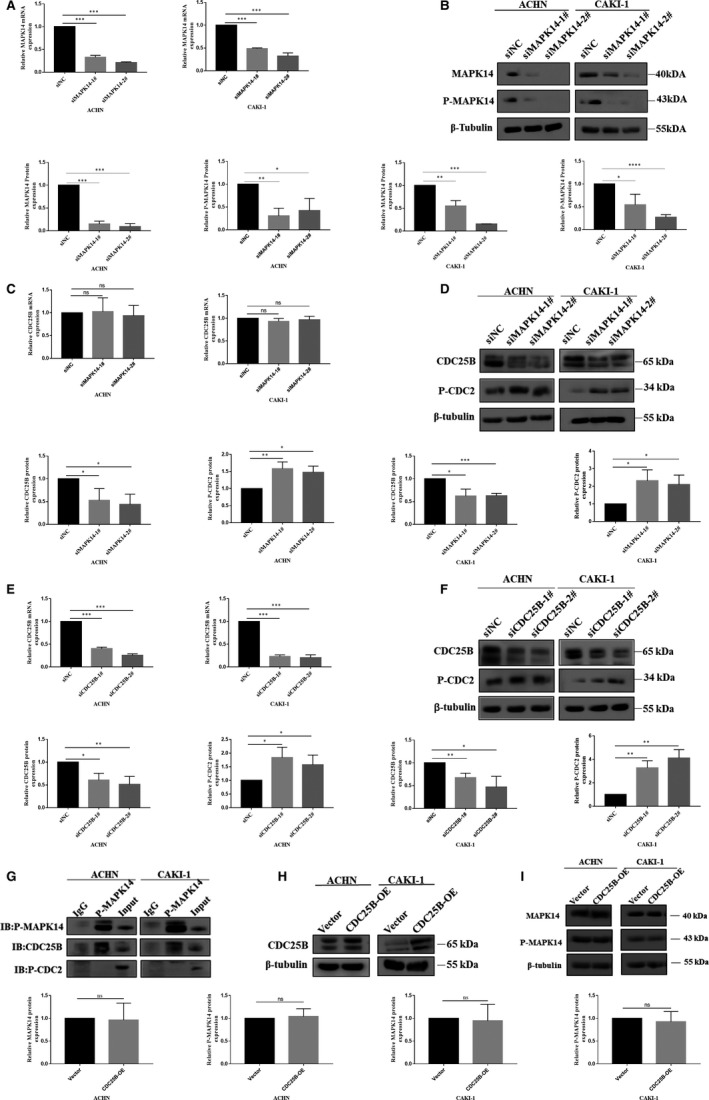
Downregulation of MAPK14 resulted in decreased expression of P‐MAPK14 and CDC25B while overexpression of CDC25B did not affect MAPK14 and P‐MAPK14. A, MAPK14 mRNA levels in ACHN and CAKI‐1 cell lines determined by RT‐PCR. B, Protein levels of MAPK14 and P‐MAPK14 measured by Western blot. C, CDC25B mRNA expression in ACHN and CAKI‐1 cells after transfection with siNC and MAPK14 siRNAs was determined by RT‐PCR. D, CDC25B and P‐CDC2 protein levels measured by Western blot. E, CDC25B mRNA levels in ACHN and CAKI‐1 cells after transfection with siNC and CDC25B siRNAs was determined by RT‐PCR. F, CDC25B and P‐CDC2 protein levels detected by Western blot. G, The interaction between P‐MAPK14 and CDC25B and P‐CDC2 was detected by CO‐IP assay. H, I, MAPK14 and P‐MAPK14 protein expression after overexpression of CDC25B in ACHN and CAKI‐1 cells was detected by Western blot. *P* < .05 was considered significant (*P < .05, **P < .01, ***P < .001), ns, not significant

To further investigate the relationship between P‐MAPK14 and CDC25B, two effective siRNAs were used to silence the mRNA of MAPK14. Subsequently, the efficiency of MAPK14 knockdown was detected by RT‐PCR and Western blot, and it was found that both the mRNA of MAPK14 and protein expression of MAPK14 and P‐MAPK14 were decreased significantly (*P* < .001, and *P* < .05, respectively) (Figure 2A,B). To investigate whether reduced protein expression of P‐MAPK14 could influence the CDC25B expression, the change in CDC25B mRNA expression after MAPK14 knockdown was examined with RT‐PCR; no significant change in CDC25B expression at the transcription level was revealed (*P* > .05) (Figure 2C). However, the decline of protein expression of CDC25B was significantly correlated with the downregulation of MAPK14 and P‐MAPK14 expressions, as shown by Western blot (*P* < .05) (Figure 2D). Surprisingly, it was found that phosphorylation of CDC2 (phospho Y15, P‐CDC2), a downstream gene of CDC25B,^22^, ^23^, ^24^ was increased (*P* < .05) (Figure 2D). To determine whether the increased P‐CDC2 was caused by the downregulation of P‐MAPK14 and CDC25B, siRNAs were used to knockdown CDC25B, and the concordant results were revealed by Western blot (*P* < .05) (Figure 2E,F). To explore the regulatory relationship of P‐MAPK14 and CDC25B with CDC2, Co‐IP experiments in two ccRCC cell lines (ACHN and CAKI‐1) were conducted. These results showed that P‐MAPK14 might directly interact with CDC25B, but not with P‐CDC2 (Figure 2G). MAPK14 and P‐MAPK14 protein levels were not found to change significantly after the overexpression of CDC25B in ccRCC cells (*P* > .05) (Figure 2H,I).

### 3.3 Knockdown of MAPK14 inhibited cell activity, proliferation, and migration

**Figure 3:**
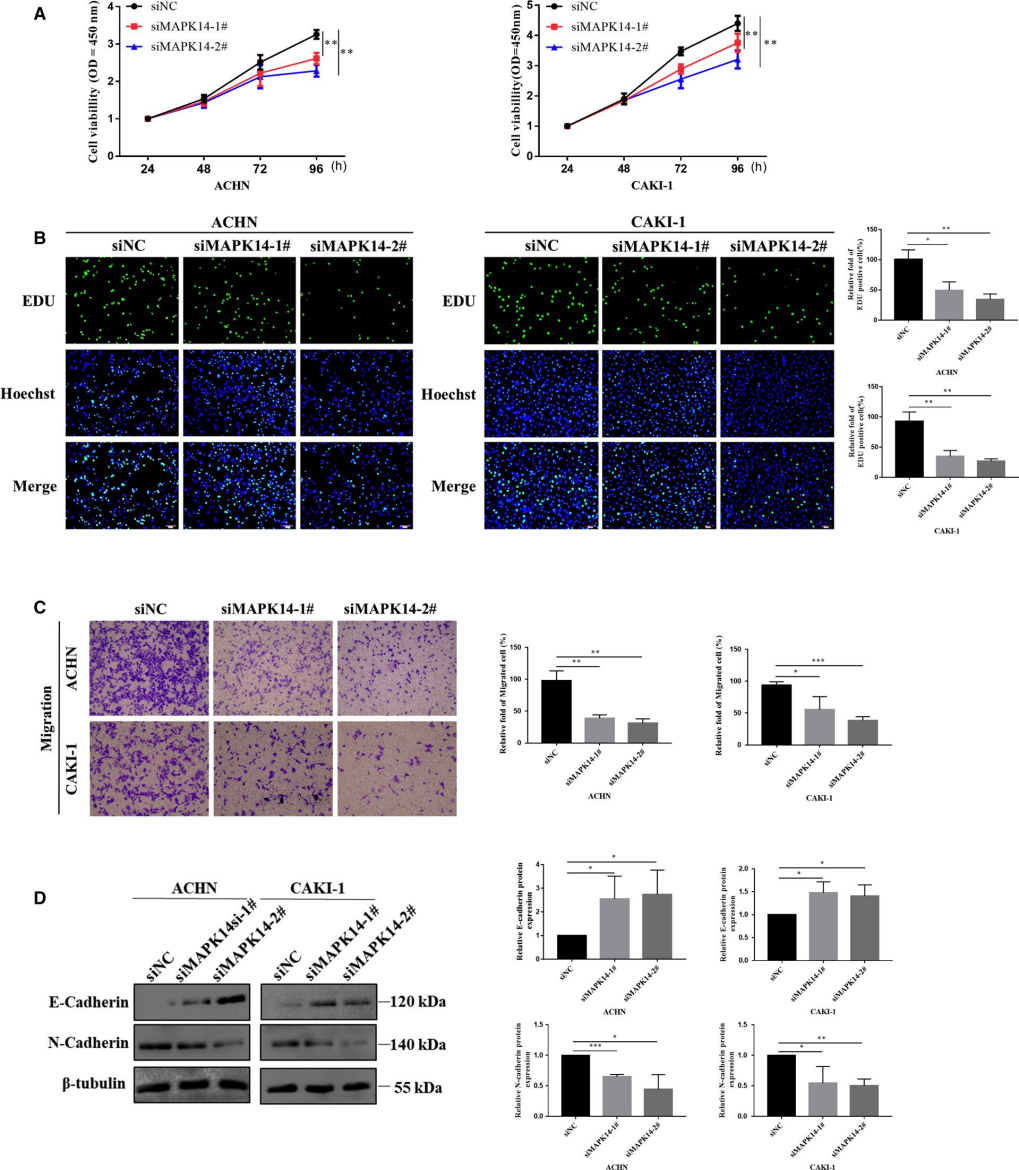
Downregulation of MAPK14 inhibited cell activity, proliferation, and migration. A, Downregulation of MAPK14 suppressed cell viability by CCK‐8 assay. B, The effect of MAPK14 knockdown on the proliferation of ACHN and CAKI‐1 cells was determined with EdU assay (magnification × 200). C, Downregulation of MAPK14 inhibited cell migration (magnification × 20). D, The protein level of cell adhesion molecules (E‐cadherin, *N*‐cadherin) detected by Western blot. *P* < .05 was considered significant (*P < .05, **P < .01, ***P < .001)

To further study the biological function of MAPK14 in ccRCC, the activity of MAPK14‐knockdown cells was analyzed by CCK‐8 assay. The results revealed that after transfection with MAPK14 siRNAs for 72 hours, the activity of ACHN and CAKI‐1 cells was significantly reduced compared with the siNC group (*P* < .01) (Figure 3A). Cell proliferation test was also conducted in these cell lines. The EdU test showed that the proliferation ability of the cells decreased significantly after MAPK14 knockdown (*P* < .05) (Figure 3B). These results suggest that knockdown of MAPK14 could inhibit the activity and proliferation ability of ccRCC cells in vitro.

To confirm whether MAPK14 was involved in cell migration, ACHN and CAKI‐1 cell lines were used to assay cell migration. After 48 hours of transfection with siMAPK14‐1# and siMAPK14‐2#, the migration ability of ACHN and CAKI‐1 cells was significantly decreased (*P* < .05) (Figure 3C). Since the upregulation of E‐cadherin and downregulation of *N*‐cadherin have been demonstrated to be related to the reduced ability of cell migration, the expressions of E‐cadherin and *N*‐cadherin after MAPK14‐knockdown were detected with Western blot to further verify this observation. When MAPK14 was silenced in these cells, the expression of E‐cadherin was significantly upregulated compared with the control group, while the expression of *N*‐cadherin was downregulated significantly (*P* < .05) (Figure 3D). These results imply that MAPK14 might play an important role in cell activity, proliferation, and migration.

### 3.4 Overexpression of CDC25B partially reversed the reduction in cell proliferation and migration caused by decreased MAPK14 and P‐MAPK14 expressions

**Figure 4:**
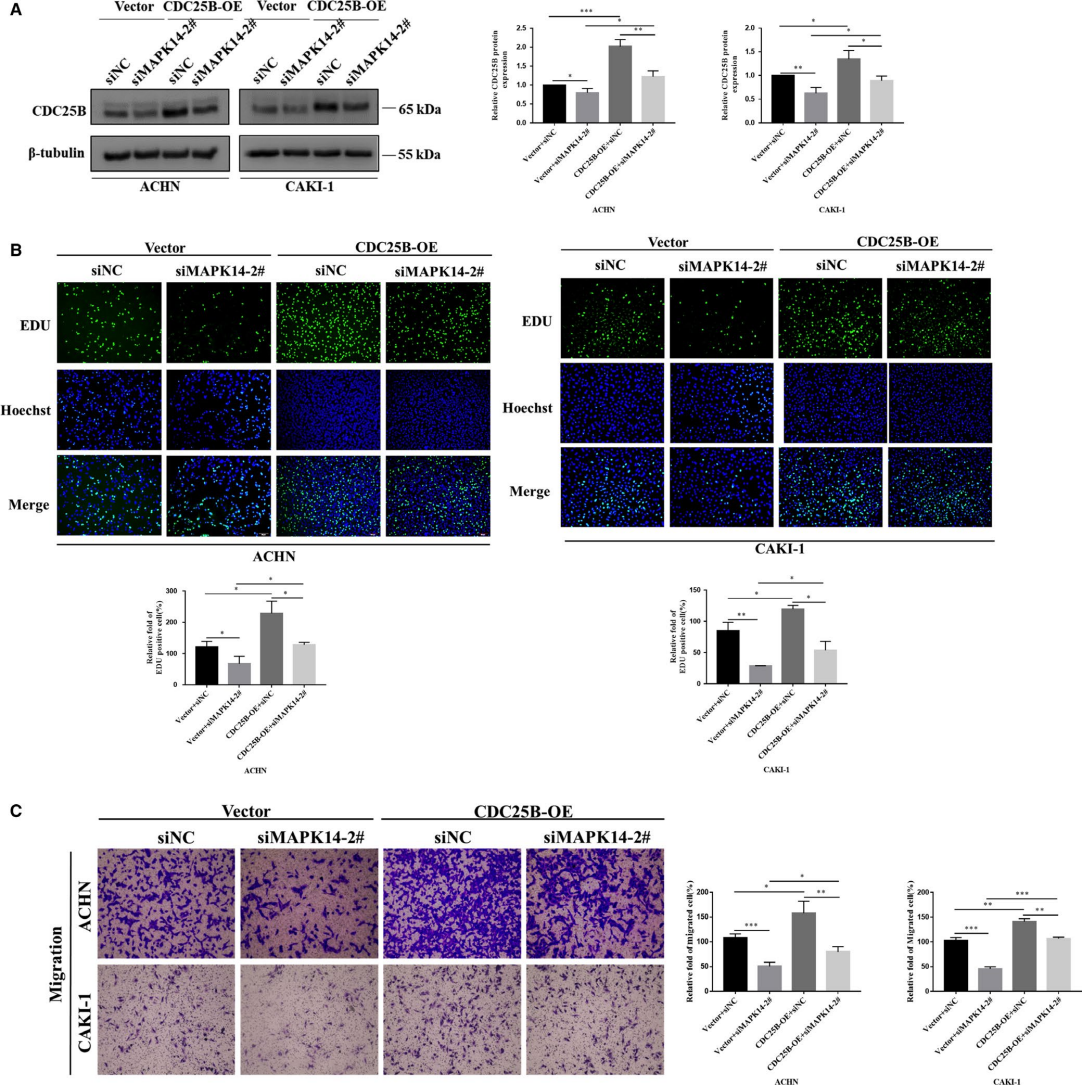
Overexpression of CDC25B could partially restore the reduction in proliferation and migration caused by decreased MAPK14 levels. A, After transfection of MAPK14 siRNAs into cells with stable high expression of CDC25B, the level of CDC25B protein was detected by Western blot. B, Proliferation of ACHN and CAKI‐1 cells was determined with EdU assay (magnification × 200). C, Transwell assay was used to examine the migration of ACHN and CAKI‐1 cells (magnification × 20). *P* < .05 was considered significant (*P < .05, **P < .01, ***P < .001)

Two stable CDC25B‐overexpression cell lines (ACHN and CAKI‐1) were constructed, then transfected with MAPK14 siRNAs. Western blot revealed that overexpression of CDC25B could partially restore the protein levels of CDC25B caused by MAPK14 silencing (*P* < .05) (Figure 4A). To investigate whether overexpression of CDC25B could reverse the decrease in cell proliferation caused by the decreased levels of MAPK14, the EdU cell proliferation assay was conducted. It was demonstrated that overexpression of CDC25B could partially restore cell proliferation (*P* < .05) (Figure 4B). Similarly, the decrease in cell migration ability caused by the reduction of MAPK14 and P‐MAPK14 was incompletely reversed as a result of CDC25B overexpression (*P* < .05) (Figure 4C). These results suggest that downregulation of MAPK1 and P‐MAPK14 might reduce the migration and proliferation of ccRCC cells in vitro, in part due to the declined levels of CDC25B.

### 3.5 MAPK14 knockdown inhibited tumor growth while CDC25B overexpression promoted tumor growth in vivo

**Figure 5:**
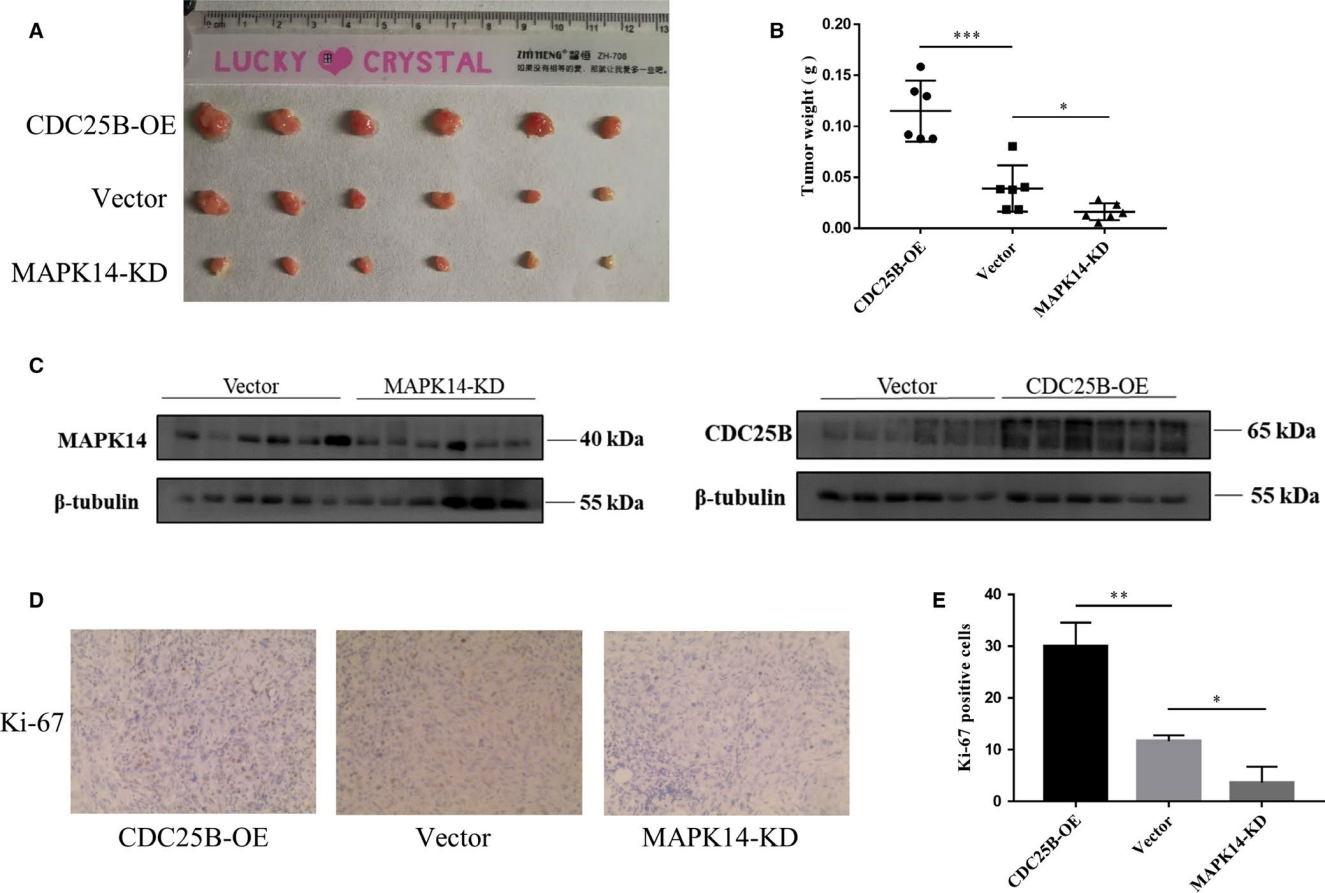
MAPK14 knockdown inhibited tumor growth and overexpressed CDC25B promoted tumor growth in vivo. A, Images of nude mice xenografts at the end of the experiment. B, Tumor weight of the three groups. C, MAPK14 and CDC25B protein levels in nude mice tumors detected by Western blot. D, IHC results revealed that compared with the vector group, the expression of Ki‐67 increased in the CDC25B overexpression group while decreased in the MAPK14 knockdown group (magnification × 40). *P* < .05 was considered significant (^*^ *P* < .05, **P < .01, ***P < .001)

To further investigate the effect of MAPK14 and CDC25B on tumor in vivo, MAPK14 knockdown and CDC25B overexpression plasmids were constructed and transfected into ACHN cell line. Stable transfected ACHN cell line was screened, and ccRCC xenotransplantation models in nude mice were established. After 4 weeks, it was found that the tumor size and weight in MAPK14 knockdown group were significantly lower than those of vector group, while in CDC25B overexpression group, the tumor size and weight were significantly higher than those of the vector group (*P* < .05) (Figure 5A,B). Compared with vector group, the protein expression of downregulation of MAPK14 group and upregulation of CDC25B group was detected by Western blot (Figure 5C). In addition, IHC analysis showed that the Ki‐67 expression was increased in the CDC25B overexpression group and was decreased in MAPK14 knockdown group (*P* < .05) (Figure 5D). These results indicate that downregulation of MAPK14 might inhibit tumor proliferation, while overexpression of CDC25B might promote tumor proliferation in vivo.

### 3.6 Related genetic analysis: MAPK14 phosphorylation

**Figure 6:**
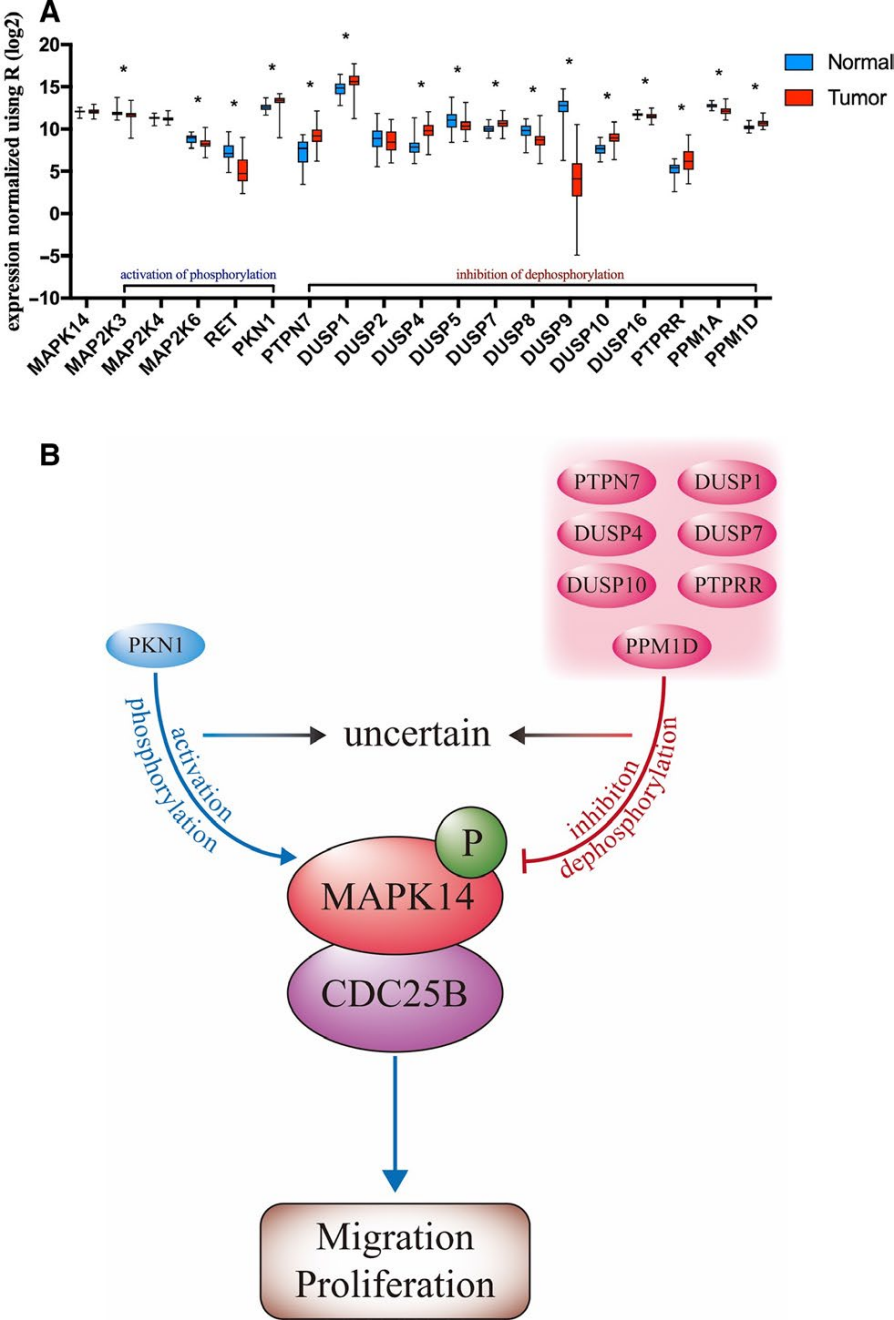
The phosphorylation‐related genes of MAPK14 and the pattern of proteins interaction in ccRCC. A, The selected genes include those that phosphorylate Thr180, Tyr182, and unlabeled sites. B, The hypothesis illustrates the interaction of MAPK14, P‐MAPK14, and CDC25B in ccRCC. *P < .05 was considered significant

In a diseasenetwork open resource, we found genes promoting phosphorylation and inhibiting dephosphorylation of MAPK14 (Figure 6A). The selected genes include those that phosphorylate both Thr180 and Tyr182, and unlabeled sites. The transcription levels of these genes were analyzed using the 72 control samples previously processed from TCGA database. Although the protein status of these genes cannot directly be inferred, it can be used as a reference for analysis. According to the comparative analysis of ccRCC and healthy tissues, PKN1, PTPN7, DUSP1, DUSP4, DUSP7, DUSP10, PTPRR, and PPM1D may maintain the phosphorylation of MAPK14. However, certain genes might participate in the posttranslational modification of MAPK14, such as MAP2K3 and MAP2K6. This result is not conclusive, but it might helpful with the future studies. Therefore, it could infer that in ccRCC, the selected genes may be involved in phosphorylation of MAPK14, and P‐MAPK14 may regulate cell migration and proliferation by binding to CDC25B and affecting its protein stability (Figure 6B).

## 4 DISCUSSION

Tens of thousands of patients with ccRCC still face the threat of death every year. Recent years, VEGF targeted drugs, mTOR inhibitors and cytokines have result in significant progress in the management of ccRCC. The immunotherapy for ccRCC has also made significant progress, such as PD‐1/PD‐L1 inhibitors. Despite these advances, important questions remain to be resolved, regarding biomarkers of efficacy, patient selection, and the optimal combination and sequencing of agents.^25^ Identification of the effective biomarkers and elucidation of the signaling processes responsible for the development and progression of ccRCC would be helpful with the exploration of potentially effective therapies.

Three distinct subfamilies of MAPKs are p38, c‐Jun N‐terminal kinase (JNK), and extracellular signal‐regulating protein kinase (ERK 1/2), with a similarity of 60%‐70%.^26^ As an important member of the p38 MAPK family, MAPK14 has been reported to play a dual role in several cancers.^9^ Some studies have demonstrated that MAPK14 promotes the occurrence and progression of breast cancer by activating its downstream target genes.^27^, ^28^ Moreover, MAPK14 has also been shown to act as a suppressor in some malignancies, such as liver, colon, and lung cancer.^29^, ^30^, ^31^ In ccRCC, it has been reported that PTEN can decrease MAPK14 activity and inhibit cell migration and proliferation, while upregulation of long noncoding RNA BX357664 can suppress the proliferation, migration, and the activity of ccRCC cells by decreasing the phosphorylation level of MAPK14^32^, ^33^. Furthermore, phosphorylation of MAPK14 has been reported to stimulate the progression or recurrence of lung, pancreatic, and colon cancers.^34^, ^35^, ^36^ However, none of these studies has directly targeted MAPK14 or P‐MAPK14 in ccRCC. In this study, there were no significant differences in both the transcription and translation levels of MAPK14 between ccRCC tissue and adjacent healthy tissue, but the level of P‐MAPK14 of ccRCC tissue was significantly higher than that of the adjacent healthy tissues.

In this study, there was no increase in the level of transcription and translation of MAPK14 in ccRCC. However, P‐MAPK14 level was markedly elevated in ccRCC tissues. In addition, the high expression of P‐MAPK14 and CDC25B proteins was closely related to the Fuhrman grade of ccRCC tissues. The expression of P‐MAPK14 was significantly associated with the expression of CDC25B at the protein level in tumor samples. After MAPK14 knockdown, there was significant decrease in the protein levels of P‐MAPK14 and CDC25B, but there was no significant change in the transcription level of CDC25B, indicating that MAPK14 could not affect the transcription of CDC25B, but could influence its protein expression. Therefore, in combination with the strong correlation between P‐MAPK14 and CDC25B, it could be hypothesized that P‐MAPK14 might impact the protein stability of CDC25B. This was confirmed by the results of Co‐IP experiments, which showed that P‐MAPK14 could directly bind with CDC25B. It has been demonstrated that p38 can bind to and phosphorylate CDC25B,^21^ and activation of p38 might phosphorylate CDC25B at Ser101, leading to the rapid degradation of CDC25B and cell cycle arrest.^37^ Here, it was found that the level of MAPK14 phosphorylation in ccRCC tissues was higher than that of the healthy tissues, and P‐MAPK14 could affect the stability of CDC25B. These results suggest that MAPK14 and P‐MAPK14 might facilitate the proliferation and metastasis of ccRCC. In addition, these results indicate that overexpression of CDC25B may not be able to influence the protein expression of MAPK14 and P‐MAPK14, which is consistent with the hypothesis that P‐MAPK14 could stabilize the CDC25B protein.

It has been reported that barbaloin treatment can inhibit the levels of P‐MAPK14 and CDC25B in non‐small cell lung carcinoma, and suppress cell proliferation and migration consequently.^38^ In this study, it was demonstrated that P‐MAPK14 and CDC25B expressions in ccRCC were significantly higher than those in adjacent healthy tissues. Both P‐MAPK14 and CDC25B could stimulate cell proliferation and metastasis in ccRCC in vitro and in vivo.

As some genes could produce certain functions after posttranslational modification. For example, phosphorylated MEK3/6 can promote phosphorylation of MAPK14^39^. To determine the upstream genes promoting MAPK14 phosphorylation, genes that phosphorylated MAPK14 at the transcription level in the 72 pairs of ccRCC and adjacent healthy tissues from TCGA database were analyzed, and it was found that many genes were highly expressed in ccRCC, which might facilitate the phosphorylation of MAPK14. Indeed, this is just an inference, requiring the verification of future studies.

The binding of P‐MAPK14 to CDC25B was confirmed in this study; however, it was not clear whether the former could directly have an impact upon the protein stability of the latter. After MAPK14 knockdown, there might be a chain reaction‐like event, which might be caused by the changes of other undiscovered factors. Collectively, P‐MAPK14 and CDC25B proteins were overexpressed in ccRCC. P‐MAPK14 could promote tumor proliferation and migration through binding to and affecting the stability of CDC25B. This study provides new perspectives and directions for the investigation of the molecular mechanisms ccRCC, which would be valuable for the development of novel therapies for ccRCC.

## CONFLICT OF INTEREST

The authors declare that they have no conflict of interests.

## AUTHOR CONTRIBUTIONS

JL and XY performed the experiments and finished the manuscript. HY and BL collected the data. ZZ analyzed the data; CK and ZL conducted experimental design. ZL supervised all experiments and manuscript. All authors have read and approved the final manuscript.

## ETHICS APPROVAL AND CONSENT TO PARTICIPATE

The Ethics Committee of the First Hospital of China Medical University (Shenyang, China) approved the use of human tissue samples for experiments. All participants provided written informed consent for the entire study. Animal experiments were performed after being approved by the Animal Ethics and Welfare Committee of China Medical University.

## PATIENT CONSENT FOR PUBLICATION

All participants provided written informed consent for the entire study.

## Data Availability

The datasets used and/or analyzed during the current study are available from the corresponding author on reasonable request.
