# Supplementary figures and images for: Knockdown of MAPK14 inhibits the proliferation and migration of clear cell renal cell carcinoma by downregulating the expression of CDC25B
Source: Cancer Med. 2019 Dec 19;9(3):1183–95. doi: 10.1002/cam4.2795 (PMC6997073; doi:10.1002/cam4.2795)

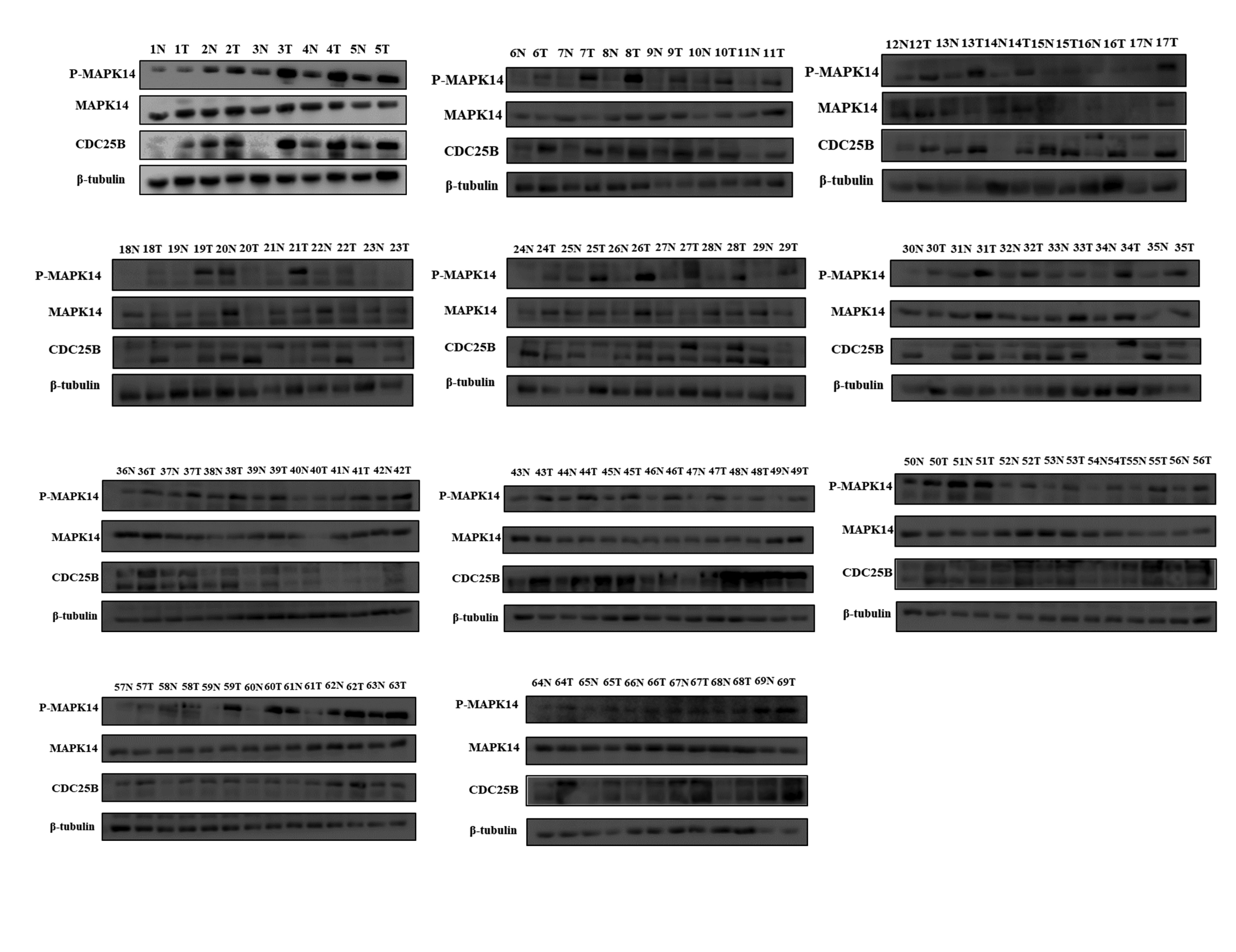

Supplement: Supplementary file 1 [file CAM4-9-1183-s001.tiff]
