# Supplementary material for: Knockdown of MAPK14 inhibits the proliferation and migration of clear cell renal cell carcinoma by downregulating the expression of CDC25B
Source: Cancer Med. 2019 Dec 19;9(3):1183–95. doi: 10.1002/cam4.2795 (PMC6997073; doi:10.1002/cam4.2795)
Supplement: Supplementary file 2 [file CAM4-9-1183-s002.docx]

Supplement figure 1.

Expression of MAPK14, P-MAPK14, and CDC25B in ccRCC and adjacent healthy tissue. 16, 17, and 61 are papillary renal cell carcinoma and the remaining 66 pairs are clear cell renal cell carcinoma.
